# Differential Apoptotic Responses of Hemocyte Subpopulations to White Spot Syndrome Virus Infection in *Fenneropenaeus chinensis*

**DOI:** 10.3389/fimmu.2020.594390

**Published:** 2020-12-07

**Authors:** Chuang Cui, Qianrong Liang, Xiaoqian Tang, Jing Xing, Xiuzhen Sheng, Wenbin Zhan

**Affiliations:** ^1^ Laboratory of Pathology and Immunology of Aquatic Animals, KLMME, Ocean University of China, Qingdao, China; ^2^ Laboratory for Marine Fisheries Science and Food Production Processes, Qingdao National Laboratory for Marine Science and Technology, Qingdao, China

**Keywords:** apoptosis, hemocyte subpopulations, *Fenneropenaeus chinensis*, white spot syndrome virus, hemocytes

## Abstract

The apoptosis of hemocytes plays an essential function in shrimp immune defense against pathogen invasions. In order to further elucidate the differential apoptotic responses of the granulocytes and the hyalinocytes in *Fenneropenaeus chinensis* post WSSV infection, the characteristics of apoptotic dynamics and viral proliferation in total hemocytes and hemocyte subpopulations were respectively investigated in the present work. The results showed that the apoptotic rate of hemocytes changed significantly, and the apoptosis-related genes also showed significantly differential expression responses during WSSV infection. Interestingly, we found that the apoptotic rate of virus-negative hemocytes was significantly higher than that of virus-positive hemocytes in the early stage of WSSV infection, while it was significantly lower than that of virus-positive cells in the middle and late infection stages. The difference of apoptosis between virus-positive and virus-negative hemocytes seems to be an important way for the WSSV to destroy the host’s immune system and facilitate the virus spread at different infection stages. It was further found that the apoptosis rate of granulocytes was always significantly higher than that of hyalinocytes during WSSV infection, indicating that granulocytes have a stronger apoptotic response to WSSV infection. Moreover, a higher viral load was detected in granulocytes, and the density of granulocytes decreased more rapidly post WSSV infection, indicating that the granulocytes are more susceptible and vulnerable to WSSV infection compared with the hyalinocytes. These results collectively demonstrated that the apoptotic response in shrimp hemocytes was significantly influenced by the WSSV infection, and the differential apoptotic response of granulocytes and hyalinocytes to WSSV indicated the differences of antiviral mechanisms between the two hemocyte subpopulations.

## Introduction

As invertebrates, shrimp lack the acquired immunity and depend on the inherent immune system to combat pathogen invasion (1, 2). Hemocyte is one of the main immune cells in shrimp, which plays crucial roles both in humoral and cellular immunity. Hemocytes participate in the humoral immunity process by synthesizing and releasing immune molecules such as antibacterial peptides, lectins, and crustin (3–5). Hemocytes are involved in the cellular immunity process mainly through phagocytosis, apoptosis, encapsulation and nodule formation (6, 7). Based on their stainability and morphological features, hemocytes of shrimp are generally divided into granulocytes, semi-granulocytes and hyalinocytes (1, 8). Some researchers separated the three types of hemocyte subpopulations through flow cytometry or Percoll gradient centrifugation, which exhibited different expression profiles of immune-related genes (9, 10). However, we found that it is extremely difficult to sort out high-purity hemocyte subpopulations of *Fenneropenaeus chinensis* by these two methods. Fortunately, the granulocytes (containing semi-granulocytes) and the hyalinocytes with purities of up to 95% were successfully separated in our previous studies by immunomagnetic bead combined with monoclonal antibodies that could specifically recognize granule-containing hemocytes (including semi-granulocytes and granulocytes) of *F. chinensis*, and the differential protein expression profiles between the two subpopulations were further explored (11–13). It was also reported that the hyalinocytes participate in the immune response mainly through phagocytosis, while the granule-containing hemocytes resist pathogens by storing and releasing immune factors, such as pro-phenoloxidase (proPO), AMPs, and others (14–16). These evidences demonstrated that those differences of the hemocyte subpopulations are the functional basis for shrimp in response to pathogen invasion.

Apoptosis, a kind of programmed cell death, plays vital roles in a variety of biological processes and diseases (17, 18). It is generally considered that apoptosis serves as a kind of innate immune pathway conserved in shrimp, involving in the antiviral process (19). When the pathogen invades the host cells, the cells initiate apoptosis to hamper the pathogen proliferation and spread (19–21). However, some pathogens have evolved diverse strategies to inhibit apoptosis, thereby providing favorable conditions for self-proliferation (22, 23). When the pathogen completed proliferation, the intentionally induced apoptosis would facilitate the release of pathogen progeny (24, 25). White spot syndrome virus (WSSV), a large enveloped DNA virus, could infect most shrimp species and has caused huge economic losses in the shrimp culture industry (26, 27). Previous studies have confirmed that WSSV infection induced apoptosis of major tissues cells in shrimp (28). Over the years, it has been proven that some apoptosis-related genes in shrimp could inhibit or promote WSSV proliferation (29, 30). Conversely, some WSSV envelope proteins could also modulate the host cell apoptosis during infection, thereby facilitating viral replication and expansion (31–34). In our previous studies, it was also found that the hemocytes apoptosis process in *Litopenaeus vannamei* fluctuated significantly during WSSV infection (35). These evidences suggested that the WSSV infection is closely related to the apoptotic process. Meanwhile, WSSV appears to exhibit different proliferation dynamics in different hemocyte subpopulations (36, 37). And WSSV infection also caused the differential protein expressions between the granulocytes and hyalinocytes of shrimp (13). These reports indicated that there are differences in immune responses among the hemocyte subpopulations during WSSV infection. However, there have been no studies on the differential apoptotic responses of hemocyte subpopulations to WSSV infection and the differences of viral infection characteristics between hemocyte subpopulations.

In order to provide the thorough comprehension of relationship between the apoptotic dynamics of hemocytes and the WSSV infection process, the TUNEL assay and real-time qPCR were utilized to detect the characteristics of hemocytes apoptosis in *F. chinensis* during WSSV infection. Meanwhile, the WSSV infection process in hemocytes and the accumulative mortality of WSSV-infected shrimp were also investigated. Moreover, the laboratory-prepared monoclonal antibodies combined with the TUNEL assay and immunomagnetic bead method were used to further study the differential apoptotic responses and the differences of WSSV-infection process between granulocytes and hyalinocytes during WSSV infection, which would provide more information on the functional differences of the hemocyte subpopulations.

## Materials and Methods

### Shrimp and WSSV Infection

The healthy shrimp (*F. chinensis*, 15–18 cm) were obtained in a Qingdao Harbor, Shandong province, China. Five shrimp were randomly selected for testing WSSV to ensure that the shrimp were WSSV-free according to the method described previously (38). The shrimp were kept in aerated seawater at 24 °C for about a week before the experiment. This study was carried out in agreement with the International Guiding Principles for Biomedical Research Involving Animals documented by Guide for the Use of Experimental Animals of the Ocean University of China. This study was also approved by the Committee of the Ethics on Animal Care and Experiments at Ocean University of China (permit number: 20180101).

In short, the gill tissue (1 g) from the heavily WSSV-infected *F. chinensis* was homogenized in 10 ml sterile prawn homoiosmotic phosphate buffered saline (PHPBS), and the homogenized solution was centrifuged at 600 ×g for 20 min and filtered using a 450 nm membrane. The filtrate was centrifuged at 55000 ×g for 1.5 h at 4°C to take the supernatant, and then the WSSV copies in inoculum was determined (39).

A total of 1400 healthy shrimp were divided into the control group and the WSSV infection group. In the WSSV infection group, 1100 shrimp were intramuscularly injected with 100 μl WSSV inoculum (containing 10^4^ copies/μl), of which 950 shrimp were used for sampling, and the remaining 150 shrimp were divided into 3 groups on average for recording mortalities. In the control group, 300 shrimp were treated with the same amount of sterile PHPBS. The 150 shrimp were utilized to the sampling, and another 150 shrimp were also equally divided into 3 groups for recording the cumulative mortalities.

### Sampling of Hemocytes

The hemocytes were collected at 0, 6, 12, 18, 24, 36, 48, 60, 72 h post infection (hpi) in the two groups, respectively. In the WSSV infection group, the hemocytes were collected from 30 shrimp and pooled as one separate sample, and a total 90 shrimp were sampled (n=3) at each sampling point. In the control group, the hemocytes of 4 shrimp were used as one separate sample, and 12 shrimp were sampled (n=3) at each time point. The modified cold Alsever solution (27 mM Nacitrate, 336 mM NaCl, 115 mM glucose, 9 mM EDTA, pH 7.2, AS) was used as anticoagulant, and the hemolymph was withdrawn from the pericardial cavity using a syringe containing the anticoagulant. Then, the collected hemocytes were centrifuged at 400 ×g for 5 min at 4 °C and washed three times with sterile PHPBS. In addition, the total hemocytes count in hemolymph was calculated as previous method (35). Briefly, 10 μl of hemocytes suspension was diluted for 10 times in 90 μl PHPBS, then the density of the diluted hemocyte suspension was measured by Neubauer hemocytometer, and THC was obtained by calculation.

### Detection of Apoptotic Hemocytes by Flow Cytometric Immunofluorescence Assay (FCIFA)

The DNA fragmentation due to cell death by apoptosis could be labeled with the TUNEL method (40). Therefore, the apoptotic hemocytes were detected by TUNEL Assay Kit (Biovision, USA) in combination with flow cytometry (11, 41). The 2% paraformaldehyde was used to fix approximately 10^6^ hemocytes for 15 min, and then rinse twice with PHPBS. The fixed hemocytes were permeabilized with 0.1% TritonX-100 for 15 min. After rinsed with PHPBS twice, the pelleted hemocytes were resuspended in the DNA fragment labeling solution and incubated at 37 °C for 1 h. The TdT-enzyme negative solutions were used as controls. Following rinsed twice and precipitated, the hemocytes were incubated in 100 μl diluted Anti-BrdU-Red antibody solution at room temperature (RT) in the dark for 25 min. After rinsed and resuspended with PHPBS, it was analyzed by flow cytometer (Accuri C6, BD Biosciences, USA).

### Detection of WSSV-Positive Hemocytes by FCIFA

The WSSV-positive hemocytes were labeled with mixed mouse anti-WSSV monoclonal antibodies (Mabs 1D5, 1G12, 3B7,) previously produced in our laboratory and analyzed by flow cytometry (35, 42, 43). The hemocytes (about 10^6^ cells in total) were fixed and permeabilized as above. Then the hemocytes suspension was incubated with mixed diluted Mabs for 1 h at 37 °C. Following three rinses with PHPBS, the hemocytes suspension was incubated with goat anti-mouse IgG Alexa Fluor®488 antibody (Invitrogen) for 45 min at 37 °C in the dark, and washed as above. Then, the hemocytes were suspended in 1 ml PHPBS and analyzed by Accuri C6 ﬂow cytometer. The myeloma culture supernatant instead of anti-WSSV Mabs to be used as the control.

### Quantification of Apoptotic-Related Genes Expression and WSSV Copies in Hemocytes by RT-qPCR

For total RNA isolation, the each hemocytes sample (about 10^5^ cells in total) was dissolved in TRIzol^®^ reagent (Invitrogen, USA) and the quality and quantity of total RNA was examined by a Nanodrop 8000 spectrophotometer (Thermo Scientific, MA, USA). The single-strand cDNA was synthesized using 2 μg DNA-free RNA by M-MLV reverse transcriptase reagent Kit (Promega, USA). Then the expression profiles of three apoptosis-related genes in hemocytes including Caspase (*Fc*Caspase), Cellular Apoptosis Susceptibility Protein (*Fc*CAS) and translationally controlled tumor protein (*Fc*TCTP) were detected by RT-qPCR with specific primers (shown in **Table 1**). The RT-qPCR was performed in triplicate using SYBR Green I Master mix (Roche, Basel, Switzerland) in a LightCycler^®^480 II Real Time PCR System (Roche, Basel, Switzerland). The procedures were performed as following: 1 cycle of 95 °C for 5 min and 40 cycles of 95 °C for 5 s, annealing for 20 s, and 78 °C for 1 s. The 18S rRNA was used as the reference gene. The relative gene expression levels were calculated by the 2^-△△t^ method.

**Table 1:** Primers used in the present study.

Primer name	Primer sequence (5′-3′)	Application
*Fc*Caspase-F	AAGGGAATCCAAGGGAGTGTC	qRT-PCR primers
*Fc*Caspase-R	ATCGGTTTCGTAGAGGACTGC	
*Fc*CAS-F	ACTTTGCCCAGCCACTTACA	qRT-PCR primers
*Fc*CAS-R	CTGGTAAGTCCTGCGAGTTGAG	
*Fc*TCTP-F	TGGCGTAATCTATGAAGTAACAGG	qRT-PCR primers
*Fc*TCTP-R	CGAAAGCATAACCTTCTTGTAGC	
18SrRNA-F	ACAATGGCTATCACGGGTAACG	Internal reference
18SrRNA-R	CTGCTGCCTTCCTTAGATGTGGTA	
QVP28F	AAACCTCCGCATTCCTGTGA	WSSV detection primers
QVP28R	TCCGCATCTTCTTCCTTCAT	

The WSSV copies in the samples were investigated according to the WSSV detection curve that established previously (39, 44). In short, the total genomic DNA of each hemocytes sample (about 10^5^ cells in total) was isolated using DNA extraction kit (Takara, Japan) and quantified by Nanodrop 8000 Spectrophotometer. Then 50 ng DNA was added into each qPCR premix with primer pairs (QVP28F and QVP28R, shown in **Table 1**) for amplification, the absolute quantification PCR was carried out and the data was analyzed as previously described (35).

### Detection of Apoptotic WSSV-Positive Hemocytes by Double Immunofluorescence Flow Cytometry and Immunofluorescent Assay (IFA)

The prepared hemocytes (containing about 10^7^ cells) sampled at 0, 6, 12, 18, 24, 36, 48, 60 and 72 hpi was incubated with the mixed anti-WSSV Mabs for 1 h at 37 °C. Following rinses, the hemocytes were incubated with the goat anti-mouse IgG Alexa Fluor^®^488 for 45 min in the dark. Then, the hemocytes were incubated with the DNA fragment labeling solution. The pelleted hemocytes were suspended in 1 ml PHPBS for analysis by Accuri C6 ﬂow cytometer.

For microscopic immunofluorescence assay, approximately 10^5^ hemocytes sampled at 48 hpi were settle onto glass slides and incubated with anti-WSSV Mabs at 37°C for 1 h. Following washing with PHPBST (PHPBS containing 0.5‰ Tween-20), the hemocytes were incubated with goat anti-mouse IgG Alexa Fluor^®^488 at 37°C for 45 min. Then the cells were rinsed and covered with DNA fragmentation labeling solution for 1 h incubation at 37°C. Following rinses, 25 μl Anti-BrdU-Red antibody solution was added at RT for 45 min incubation. Finally, the cells were stained with 4,6-diamidino-2-phenylindole dihydrochloride (DAPI). The hemocytes were observed under a fluorescence microscope (Olympus DP70, Japan).

### Calculation of Granulocytes and Hyalinocytes Densities by FCIFA

The density of each hemocyte subpopulation was calculated by flow cytometer. The previously produced Mabs (1G8 and1H11) that could specifically recognize the granulocytes of *F. chinensis* were used to stain the granulocytes (11, 45, 46). The hemocytes (containing about 10^6^ cells) sampled at 0, 12, 24, 36, 48, 60 and 72 hpi were incubated with diluted anti-granulocytes Mabs at 37°C for 1 h. Following rinsed, the hemocytes suspension was incubated with Alexa Fluor^®^488 antibody for 45 min at 37°C in dark. Then, the hemocytes were resuspended for calculating the density of each hemocyte subpopulation by Accuri C6 ﬂow cytometer.

### Detection of Apoptotic Granulocytes and Hyalinocytes by Double Immunofluorescence Flow Cytometry and IFA

The dynamic change of the apoptotic different hemocyte subpopulations were analyzed by flow cytometry. The pretreated hemocytes (approximately 10^7^ cells) were first incubated with the anti-granulocytes Mabs and the Alexa Fluor^®^488 as described above. After that, the hemocytes were incubated with DNA fragment labeling solution and Anti-BrdU-Red antibody solution. The hemocytes suspension were analyzed by ﬂow cytometer. For microscopic immunofluorescence assay, the hemocytes (approximately 10^5^ cells) sampled at 48 hpi were fixed and settled onto slides. The hemocytes were successively incubated with anti-granulocytes Mabs and Alexa Fluor^®^488. The hemocytes were sequentially covered with DNA fragmentation labeling solution and then with the Anti-BrdU-Red antibody solution. The cells were stained with DAPI. The apoptotic granulocytes and hyalinocytes were observed by fluorescence microscope.

### Detection of the WSSV-Positive Granulocytes and Hyalinocytes by Double Immunofluorescence Flow Cytometry and IFA

The anti-WSSV Mabs were first fluorescently labeled by Alexa Fluor^®^488 Microscate Protein Labeling Kit (life technologies, USA). 100 mg of anti-WSSV Mabs was mixed with Alexa Fluor 488 fluorescein at 1:2 (w/w) and labeled for 2 h. The mixture was filtered through a resin column to remove the remaining fluorescein and it was confirmed by fluorescence observation whether the antibody was successfully labeled with fluorescein. The WSSV-infected states of different hemocyte subpopulations were analyzed by double immunofluorescence flow cytometry. The pretreated hemocytes (approximately 10^7^ cells) sampled at 0, 12, 24, 36, 48, 60 and 72 hpi were consecutively incubated with anti-granulocytes Mabs and Alexa Fluor^®^647. Then, the hemocytes were incubated with the fluorescent-labeled anti-WSSV Mabs. The hemocytes were rinsed and resuspended for analysis by ﬂow cytometer. For microscopic immunofluorescence assay, the previously treated hemocytes were sequentially incubated with anti-granulocytes Mabs, the Alexa Fluor^®^647 and Alexa Fluor 488 labeled anti-WSSV Mabs. After DAPI staining, the hemocytes were observed by the fluorescence microscopy.

### Quantification of WSSV Copies in Granulocytes and Hyalinocytes by RT-qPCR

The different hemocyte subpopulations sampled at 0, 12, 24, 36, 48, 60, 72 hpi were sorted by immunomagnetic bead according to the method established by our laboratory, and then the numbers of WSSV copies in each hemocyte subpopulation were measured as described above (11, 44). Brieﬂy, the hemocytes (about 10^8^ cells) at each time point were incubated with anti-granulocytes Mabs. Followed rinsed with magnetic activated cell sorting buffer (MACS), the hemocytes were then incubated with goat anti-mouse IgG magnetic beads (Miltenyi Biotec, Germany). The hemocytes ﬂowed through a LS column (Miltenyi Biotecc, Germany), and collected. The collected granulocytes (magnetically labeled cells) and hyalinocytes (magnetically unlabeled cells) were separately purified by the new LS column. After labelling with goat anti-mouse IgG Alexa Fluor^®^488, the purity of sorted hemocyte subpopulation was analyzed by flow cytometry. The total genomic DNA of granulocytes and hyalinocytes were extracted and the WSSV copies in each hemocyte subpopulation sample was then determined by RT-qPCR according to the previous protocol and the results were analyzed by the established standard curve.

### Statistical Analysis

The statistical analysis was performed using software SPSS (Version 20.0; SPSS, Inc), and statistical comparisons was performed with independent-samples t test. Data were given as arithmetic mean ± S.D., and the *p*<0.05 were considered differences significant.

## Results

### The Change of Hemocyte Density and Shrimp Accumulative Mortality

**Figure 1:**
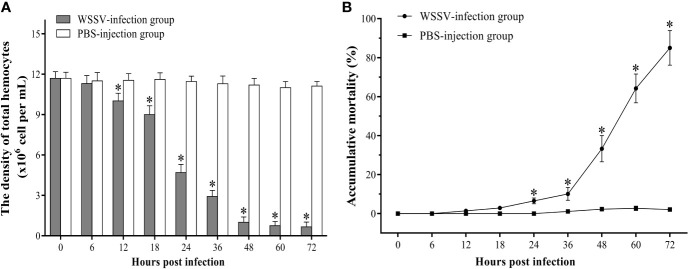
The change of the hemocytes density **(A)** and accumulative mortality **(B)** of *F. chinensis* post WSSV infection. All values are expressed as arithmetic means ± SD (n = 3). The asterisks indicate significant difference between WSSV-infection group and the control group (*p* < 0.05).

The density of total hemocytes begun to decline significantly since 12 hpi and dropped sharply to about 6% of the original hemocytes density at 48 hpi. The density of hemocytes dropped to the minimum value of 0.66±0.36×10^6^ cell/ml at 72 hpi (**Figure 1A**). The statistical results of the accumulative mortality in shrimp were shown in **Figure 1B**. Only three shrimp died in the control group. In contrast, the mortality of shrimp in the experimental group increased slightly within 36 hpi, and then increased sharply. At 72 hpi, the mortality was as high as 85%.

### The Apoptotic Process of Hemocytes and Expression Profiles of Apoptosis-Related Genes

**Figure 2:**
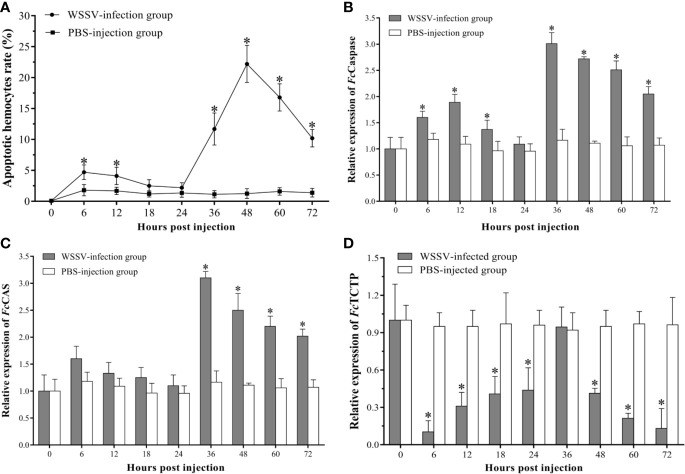
The apoptosis dynamic of hemocytes in *F. chinensis* post WSSV infection. **(A)** Analysis of the apoptotic hemocytes rates by flow cytometry. **(B–D)** qRT-PCR analysis of the expression levels of three apoptosis-related genes in hemocytes. All values are given as arithmetic mean ± S.D (n=3). Significant differences are marked with asterisks, *p < 0.05*.

The apoptotic hemocytes rate of shrimp in the infection group increased significantly and peaked at 6 hpi with an apoptotic rate of 4.7±1.2%, then decreased slightly. At 18 hpi and 24 hpi, there was no significant difference in the statistical results of apoptotic rates between the WSSV-infected group and the control group. From 24 hpi, the apoptotic rate of hemocytes in the WSSV-infected shrimp increased rapidly, and reached the second peak level with 22.2±3.0% at 48 hpi, then decline (**Figure 2A**).

Compared with the control group, the expression profile of the three apoptosis-related genes of hemocytes in the experimental group changed dramatically. The *Fc*Caspase was significantly up-regulated since 6 hpi, then decreased slightly, whereas no significant difference was found between the two groups at 24 hpi. Subsequently, the expression of *Fc*Caspase in the experimental group increased rapidly, and the peak level occurred at 36 hpi, then exhibited a slight down-regulation (**Figure 2B**). As the apoptosis-inducing gene, the expression of *Fc*CAS in WSSV infection group exhibited a slight up-regulated expression within 24 hpi, but there was no significant difference compared with the control group. However, the *Fc*CAS began to up-regulate since 24 hpi, and the peak level occurred at 36 hpi then underwent a gradual down-regulation (**Figure 2C**). The expression of the apoptotic inhibitor, TCTP, was notably lower than that of the PHPBS-injection group during the whole experimental period except for the time point of 24 hpi (**Figure 2D**).

### WSSV Proliferation in Hemocytes

**Figure 3:**
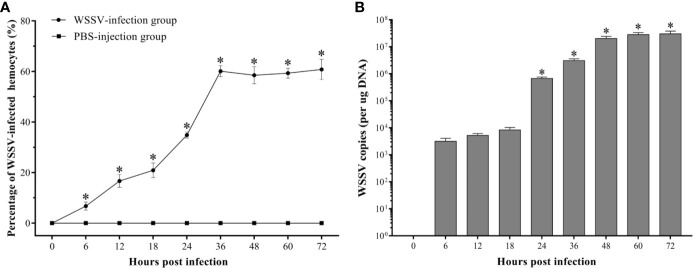
Analysis of the virus-positive hemocytes in *F. chinensis* post WSSV infection by flow cytometry and absolute qRT-PCR. **(A)** The dynamic changes of the percentage of WSSV-positive hemocytes. **(B)** Absolute qPCR analysis of the WSSV proliferation in hemocytes. The asterisks indicate significant difference when compared with the 0 hpi (*p < 0.05*). All values are given as arithmetic mean ± S.D (n = 3).

The WSSV-positive hemocytes significantly increased since 6 hpi (*p<0.05*), and reached the peak with an infection rate of 60.1±2.2% at 36 hpi, then maintained at a high level afterwards (**Figure 3A**). And the absolute qPCR assay showed that the WSSV copies did not show a significant increase within 18 hpi, but which significantly increased at 24 hpi and reached a much higher level at 48 hpi, and then maintained afterwards (**Figure 3B**).

### The Dynamic Change of Apoptotic Rate in WSSV-Positive and WSSV-Negative Hemocytes

**Figure 4:**
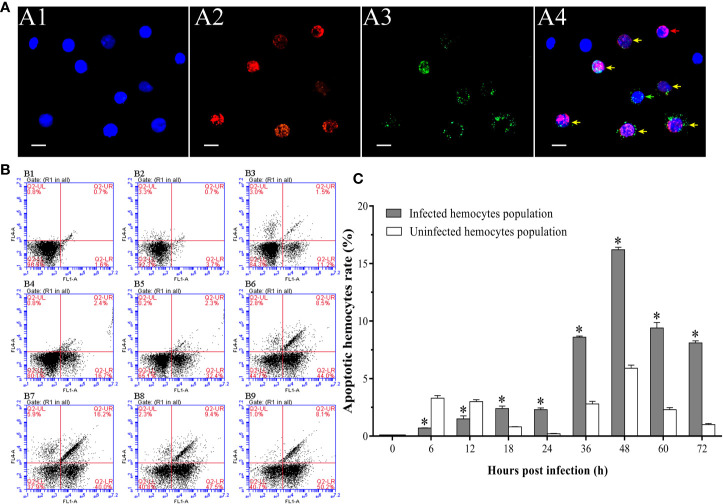
Analysis of the apoptosis dynamic of WSSV-positive and WSSV-negative hemocytes by IFA and flow cytometry. **(A)** IFA analysis of apoptotic and/or WSSV-positive hemocytes sampled at 48 hpi. A1: DAPI stained the nucleus of hemocytes; A2: TUNEL stained apoptotic hemocytes; A3: Alexa Fluor^®^488 stained the WSSV-positive hemocytes; A4: The merge of A1, A2 and A3. Bars=10 μm. The yellow arrows indicate apoptotic and WSSV-infected hemocytes; the red arrows indicate apoptotic WSSV-uninfected hemocytes; the green arrows indicate non-apoptotic WSSV-infected hemocytes. **(B)** Representative dot plots showing apoptotic rates of WSSV-infected and WSSV-uninfected hemocytes at 0, 6, 12, 18, 24, 36, 48, 60 and 72 hpi. Q2-LL: non-apoptotic and WSSV-uninfected hemocytes; Q2-LR: non-apoptotic and WSSV-infected hemocytes; Q2-UL: apoptotic and WSSV-uninfected hemocytes; Q2-UR: apoptotic and WSSV-infected hemocytes. **(C)** The apoptotic rates of WSSV-infected and WSSV-uninfected hemocytes. All values are given as arithmetic mean ± S.D (n = 3). Significant differences are marked with asterisks, *p < 0.05*.

It was observed by fluorescence microscope that the WSSV-positive hemocytes underwent different degrees of apoptosis. The most of hemocytes were observed to be apoptotic-positive and WSSV-positive cells with red and green fluorescence, while a small number of hemocytes appeared to be only apoptotic-positive cells with red fluorescent signal or only WSSV-positive cells with green fluorescent signal (**Figure 4A**). The results of double immunofluorescence flow cytometry showed that the apoptotic rate of WSSV-positive hemocytes had a slight increase within 12 hpi, but which was lower than WSSV-negative hemocytes. Subsequently, the apoptotic rate of uninfected hemocytes rapidly declined from 12 hpi to 24 hpi, while the apoptotic rate of infected hemocytes maintained at the level of 2.3±0.2% at 18 hpi and 24 hpi, which were significantly higher than that of uninfected hemocytes. Since 24 hpi, the apoptotic rates of uninfected and infected hemocytes both significantly increased and reached their respective peak values of 5.9±0.3% and 16.2±0.2% at 48 hpi, and underwent a decline afterwards. It was worth noting that the apoptotic rate of WSSV-positive hemocytes was significantly higher that of WSSV-negative hemocytes during this period (**Figures 4B, C**).

### The Time-Course Changes of the Densities and Apoptotic Rates of Granulocytes and Hyalinocytes

**Figure 5:**
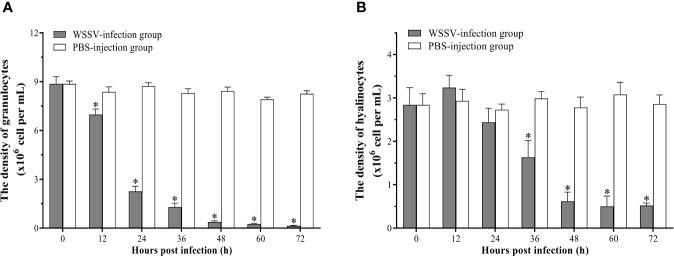
Analysis of the densities of granulocytes and hyalinocytes in hemolymph post WSSV infection by flow cytometry. **(A)**Granulocytes. **(B)** Hyalinocytes. All values are given as arithmetic mean ± S.D (n = 3). Significant differences are marked with asterisks, *p < 0.05*.

The densities of granulocytes and hyalinocytes both exhibited a significant decreasing post WSSV infection, but with different decreasing rates. Since 12 hpi, the density of granulocytes underwent a rapid decline, and decreased to 0.14±0.03×10^6^ cells per ml at 72 hpi (**Figure 5A**). Different from granulocytes, the density of hyalinocytes showed a more gradual and later decline, which was significantly decreased at 36 hpi, and then descend to 0.52±0.06×10^6^ cells per ml at 72 hpi (**Figure 5B**).

**Figure 6:**
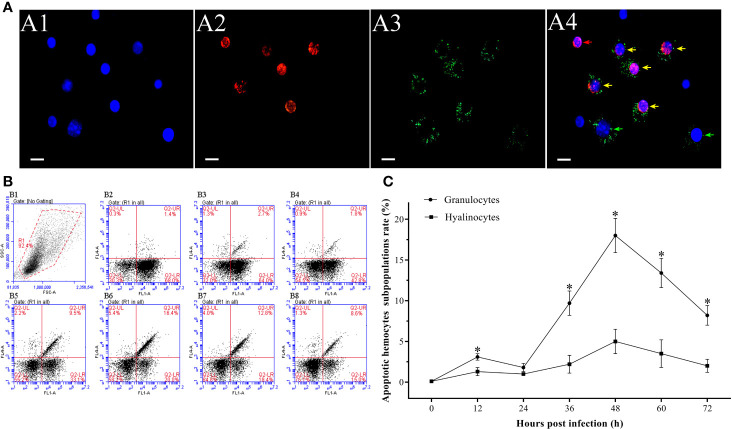
Analysis of the apoptosis dynamics of granulocytes and hyalinocytes post WSSV infection by flow cytometry and IFA. **(A)** IFA analysis of apoptotic hemocyte subpopulations sampled at 48 hpi. A1: DAPI stained the nucleus of hemocytes; A2: TUNEL stained apoptotic hemocytes; A3: Alexa Fluor^®^488 stained granulocytes; A4: The merge of A1, A2 and A3. Bars=10 μm. The yellow arrows indicate apoptotic granulocytes; the green arrows indicate non-apoptotic granulocytes; the red arrows indicate apoptotic hyalinocytes. **(B)** B1: Hemocytes were gated (R1) on FSC/SSC dot plots. B2-B8: Representative dot plots showing apoptotic rates of different hemocyte subpopulations at 0, 12, 24, 36, 48, 60 and 72 hpi. Q2-LL: non-apoptotic hyalinocytes; Q2-LR: non-apoptotic granulocytes; Q2-UL: apoptotic hyalinocytes; Q2-UR: apoptotic granulocytes. **(C)** The apoptotic rates of granulocytes and hyalinocytes. All values are given as arithmetic mean ± S.D (n = 3). Significant differences between the two hemocyte subpopulations are marked with asterisks, *p <0.05*.

In the hemocytes sampled at 48 hpi, it was observed that the number of apoptotic granulocytes with green and red fluorescent signals were significantly more than the apoptotic hyalinocytes with only red fluorescent signal (**Figure 6A**). According to the result of double immunofluorescence flow cytometry, the apoptotic rates of granulocytes and hyalinocytes increased within 12 hpi, while the apoptotic rate of granulocytes was significantly higher than that of hyalinocytes. At 24 hpi, the apoptotic rates of granulocytes and hyalinocytes were both underwent slight decrease. The apoptotic rates of granulocytes and hyalinocytes both showed rapid increases since 24 hpi, and reached their respective peak values of 18.0±2.1% and 4.0±1.5% at 48 hpi, then declined gradually. During the whole WSSV infection period, the granulocytes generally exhibited significantly higher apoptotic ratios than the hyalinocytes (**Figures 6B, C**).

### Proliferation Dynamic of WSSV in Granulocytes and Hyalinocytes

**Figure 7:**
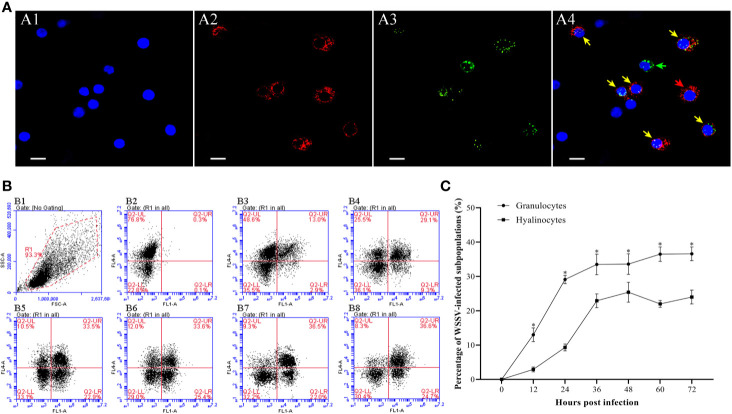
Analysis of the infection rates of granulocytes and hyalinocytes post WSSV infection by flow cytometry and IFA. **(A)** IFA analysis of WSSV-infected hemocyte subpopulations sampled at 48 hpi. A1: DAPI stained the nucleus of hemocytes; A2: Granulocytes stained with Alexa Fluor^®^647; A3: WSSV-positive cells stained with anti-WSSV monoclonal antibody labeled Alexa Fluor^®^488; A4: The merge of A1, A2 and A3. Bars=10 μm. The yellow arrows indicate WSSV-positive granulocytes; the red arrows indicate WSSV-negative granulocytes; the green arrows indicated WSSV-positive hyalinocytes, and cells without arrows were WSSV-negative hyalinocytes. **(B)** B1: Hemocytes were gated (R1) on FSC/SSC dot plots. B2-B8: Representative dot plots showing the percentages of WSSV-positive granulocytes and hyalinocytes at 0, 12, 24, 36, 48, 60 and 72 hpi. Q2-LL: WSSV-negative hyalinocytes; Q2-LR: WSSV-positive hyalinocytes; Q2-UL: WSSV-negative granulocytes; Q2-UR: WSSV-positive granulocytes. **(C)** The percentages of WSSV-positive granulocytes and hyalinocytes. All values are given as arithmetic mean ± S.D (n=3). Significant differences between the two hemocyte subpopulations are marked with asterisks, *p<0.05*.

**Figure 8:**
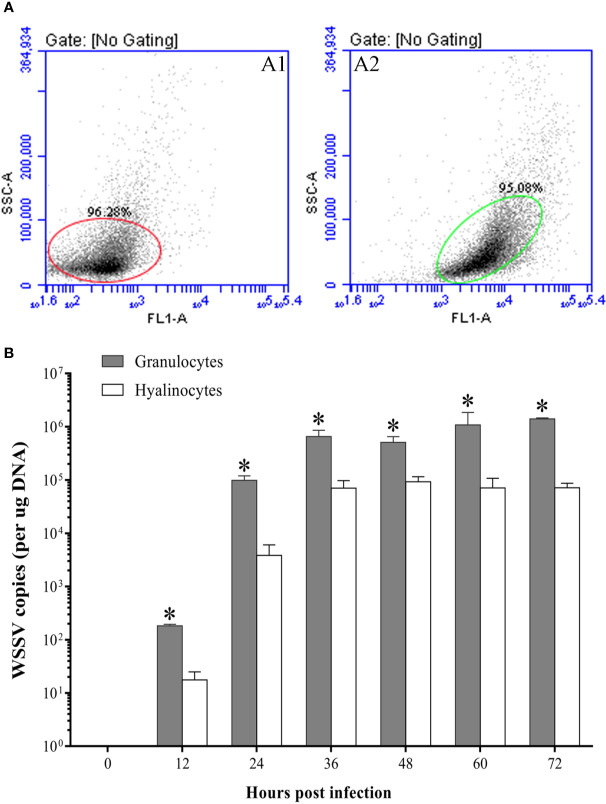
The WSSV proliferation in granulocytes and hyalinocytes. **(A)** The purities of hyalinocytes and granulocytes after sorting by flow cytometry. A1: Hyalinocytes; A2: Granulocytes. **(B)** Absolute qPCR analysis of the WSSV proliferation in hyalinocytes and granulocytes. The asterisks indicate significant difference between two hemocyte subpopulations (*p<0.05*). All values are given as arithmetic mean ± S.D (n=3).

At 48 hpi, more WSSV-positive granulocytes were observed than WSSV-positive hyalinocytes by fluorescence microscopy (**Figure 7A**). According to the result of flow cytometry, the percentage of WSSV-positive granulocytes raised from 12 hpi, and reached a value of 33.6±3.2% at 36 hpi, then were kept in the high level (**Figures 7B, C**). In contrast, the percentage of WSSV-positive hyalinocytes were always significantly lower than that of granulocytes during WSSV infection, which reached the peak value of 25.4±2.9% at 48 hpi (**Figure 7C**). Meanwhile, the immunomagnetic bead sorting was used for separating the two types of hemocyte subpopulations, and the purities of sorted granulocytes and hyalinocytes both reached more than 95% (**Figure 8A**), in which the WSSV copies were detected. According to the results of absolute qPCR assay, WSSV copies in both granulocytes and hyalinocytes increased rapidly since 24 hpi, and attained their peak levels at 36 hpi. It should be noticed that during WSSV infection, the numbers of WSSV copies in granulocytes was always significantly higher than that in hyalinocytes (**Figure 8B**).

## Discussion

As early as the 1990s, it was found that various tissues of WSSV-infected shrimp underwent varying degrees of apoptosis. And apoptosis was thought to be involved in the WSSV pathogenesis, which might be one of the main causes of death in shrimp post WSSV infection (47, 48). In another study, hemocytes apoptosis induced by yellow head virus (YHV) was also considered to be one of the main lethal causes for the infected *Penaeus monodon* (49). In the present work, the apoptotic rate of hemocytes in *F. chinensis* showed a significant increase post WSSV infection, and the total hemocytes density decreased markedly within 24 h hpi. Since 36 hpi, the apoptotic rate of hemocytes exhibited a rapid and significant increasing, then the density of hemocytes dropped rapidly and the shrimp had begun to die in large numbers. At 72 hpi, the density of hemocytes had been reduced to about 6% of normal level and the cumulative mortality of shrimp was as high as 85%. These results indicated that the apoptosis of hemocytes has a close relationship with the death of shrimp post WSSV infection, which leads to a sharp decline in density of hemocytes and severely impaired normal physiological functions of shrimp.

As one of the major ways of programed cell death, apoptosis plays the vital role during virus infection (50). Apoptosis of virus-infected cells is one of the most primitive response of organisms to viral infection. Once the invaded virus was detected, apoptosis would be quickly triggered to eliminate virus-infected cells, thereby preventing the multiplication and spreading of viruses (19, 51). For example, apoptosis could effectively prevent the spreading of virus in the early stage of hepatitis B virus infection, and it is also considered that the apoptosis is the main defense mechanism to inhibit virus replication during the influenza A virus infection (52, 53). However, studies have found that viruses have developed strategies to evade or prevent apoptosis, thereby allowing themselves to proliferate or to promote persistent infection (51). For human cytomegalovirus, myxoma virus and Epstein-Barr virus, a variety of anti-apoptotic proteins have been found to inhibit premature host cell apoptosis for facilitating viral multiplication (54–56). Meanwhile, viruses could also induce host cell apoptosis to decomposite the infected cells, thereby favoring dissemination, such as feline calicivirus, oncolytic reovirus, and porcine reproductive and respiratory syndrome virus (51, 57–59). WSSV also appears to be able to manipulate apoptosis of the host cell to provide itself with conditions for further invasion (60). Some proteins of WSSV have been found to have effects of inhibiting or promoting apoptosis in host cells during different infection stages, such as WSSV449 (also known as ORF390 or AAP-1), WSSV222, and ICP11 (61–63). WSSV449 could bind to Caspase to negatively regulate its activity and directly inhibit apoptosis. WSSV222 could inhibit apoptosis via degradation of the tumor suppressor-like protein (TSL, pro-apoptotic protein). Conversely, ICP11 is capable of binding to histones in the host cells to induce apoptosis at the late stage of WSSV infection. In this study, the apoptotic hemocytes rate increased rapidly at 6 hpi. During this period, Caspase and pro-apoptotic gene CAS were also significantly up-regulated, while the apoptosis-inhibiting gene TCTP was markedly down-regulated. The similar results were also found in our previous study in *Litopenaeus vannamei* (35). The results suggested that the hemocytes might exert antiviral effects through activating apoptosis during this stage. From 12 hpi to 24 hpi, the apoptotic rate showed a slight decrease, and the expression levels of pro-apoptotic genes also declined, while the expression of apoptotic inhibiting gene increased slightly. The percentage of WSSV-infected hemocytes increased significantly during this period. These results suggested that WSSV might inhibit apoptosis by manipulating apoptosis-related genes of host cells (34, 64). Since 24 hpi, the WSSV copies in hemocytes increased significantly, indicating that WSSV had completed replication (65). And the apoptotic rate of hemocytes also increased sharply, as did the expression of pro-apoptotic genes. During this period, apoptosis seems to be utilized by WSSV to release its progeny virus (32, 66). Consistent with previous studies, the virus might manipulate a delicate balancing act between inhibition and induction of apoptosis in host cells to facilitate their infections (67).

Interestingly, our results showed that the apoptotic rate of WSSV-negative hemocytes was significantly higher than that of WSSV-positive hemocytes within 12 hpi, whereas which subsequently had a slight decline to a level lower than that of WSSV-positive cells. Since 24 hpi, the apoptotic rates of WSSV-negative and WSSV-positive hemocytes began to rise and both peaked at 48 hpi, then decreased. These results are consistent with our previous finding of WSSV-induced hemocytes apoptosis in *L. vannamei* (35). Similarly, when WSSV infected *Penaeus monodon* and *Penaeus japonicas*, apoptosis was firstly observed in the cells without WSSV particles (28, 68). Virus-induced apoptosis of virus-negative cells could lead to host immunodeficiency and damage the host immune system (69). The apoptosis of uninfected T cells in the early HIV (human immunodeficiency virus) infection stage are the main cause of the decline in host immunity (70). And the first to be apoptotic is the viral-negative cells when the host is infected with Herpes Simplex Virus (HSV) and measles virus, then the host immune system is destroyed (71, 72). Meanwhile, the apoptosis of virus-positive cells is one of the essential immune defense strategies of organism to restrict virus replication in the initial stage of viral invasion, which has been fully confirmed (73, 74). We speculated that in the early infection stage, WSSV caused apoptosis in WSSV-negative hemocytes to weaken shrimp immunity, providing conditions for its own proliferation. During this period, apoptosis of WSSV-positive hemocytes was a critical antiviral response to eliminate the invaded virus. In the middle and late infection stages, the virus-induced apoptosis would accelerate the virus-infected hemocytes to release the progeny virus and facilitated the spreading of WSSV.

The hemocyte subpopulations were demonstrated to not only have their specifically morphological features but also play distinct roles in immunity. Granulocytes with highly refractive granules contain components of the prophenoloxidase (proPO) system, which mainly exhibit cytotoxic effects on invading pathogens. They have also proven to be the main sites for the storage and release of antimicrobial peptides and lectins. The hyalinocytes are considered the main phagocytes (15, 75). It was reported that hemocyte subpopulations also had differential responses to pathogen invasion. When spiroplasma infected *Macrobrachium rosenbergii*, the count of granulocytes increased sharply, showing a much stronger response (76). The degranulation effect of granulocytes in *L. vannamei* was significantly increased during Gram-negative bacteria infection, and the content of proPO in hemolymph was increased to resist bacterial invasion (77). The YHV infection could cause a more severe response of proteins expression profiles in granulocytes of black tiger shrimp (78). Similarly, it was also found in our previous studies that WSSV infection caused stronger protein responses in granulocytes of *F. chinensis* (13). In the present work, compared with hyalinocytes, the granulocytes showed a more rapid and significant decreasing post WSSV infection, and also had a significantly higher apoptotic rate, which indicated that granulocytes were responding more actively to WSSV infection. While the apoptotic rate of hyalinocytes did not show significant changes during early infection stage, and a slight increase in density of hyalinocytes might be due to a phagocytosis requirement in response to WSSV infection (75). Since 36 hpi, the apoptotic rates of granulocytes and hyalinocytes both increased significantly, whereas the apoptotic rate of granulocytes during this period was significantly higher than hyalinocytes, and the density of the granulocytes also decreased more severely. These results suggested that granulocytes were more vulnerable to WSSV infection and exhibited stronger apoptotic response compared with hyalinocytes during WSSV infection.

Hemocyte subpopulations seem to be differentially susceptible to pathogens invasion and proliferation. It was found that the adhesion protein for *Vibrio alginolyticus* was mainly expressed in granulocytes of *L. vannamei*, indicating the granulocytes were more susceptible to *V. alginolyticus* adhesion (79). In crayfish, the key invasive proteins including peroxinectin and heat shock protein also exhibited a much higher expression in response to *Spiroplasma eriocheiris* infection (80). The yellow head virus was detected to be mainly proliferate in the granulocytes of black tiger shrimp (78). WSSV has previously been confirmed to propagate primarily in the granulocytes of *Penaeus merguiensis* (36). In the present work, we also found that the percentage of WSSV-positive granulocytes and the numbers of WSSV copies in granulocytes were always notably higher than that of hyalinocytes during the WSSV infection process, which indicated that granulocytes were more susceptible to WSSV infection. The differential susceptibility to WSSV might also be an important reason for the differential apoptotic responses between the two types of hemocyte subpopulations.

In summary, we found that hemocytes apoptosis might play different roles in the different WSSV infection stages. WSSV infection induced differential apoptotic responses between granulocytes and hyalinocytes, and granulocytes appeared to be more susceptible to WSSV infection and exhibited stronger apoptotic responses. The relevant results fully confirmed the close relationship between the pathogenicity and lethal mechanism of WSSV and apoptosis. This research would deepen our thorough comprehension of the intimate relationship between apoptosis in hemocytes and WSSV infection, and facilitate us to understand the functional differences of hemocyte subpopulations in innate immunity of shrimp.

## Data Availability Statement

The raw data supporting the conclusions of this article will be made available by the authors, without undue reservation.

## Ethics Statement

The animal study was reviewed and approved by the Committee of the Ethics on Animal Care and Experiments at Ocean University of China.

## Author Contributions

CC and QL designed the study, performed experiments and statistically analyzed the results, and drafted and revised the manuscript. XT guided and designed the study, and drafted and revised the manuscript. JX and XS provided research thought and helped experimental data processing. WZ provides research thought and provides reagents and experimental space. All authors contributed to the article and approved the submitted version.

## Funding 

The research was supported by the National key Research and Development Program of China (2018YFD0900504), Qingdao National Laboratory for Marine Science and Technology (QNLM2016ORP0307) and the Taishan Scholar Program of Shandong Province.

## Conflict of Interest

The authors declare that the research was conducted in the absence of any commercial or financial relationships that could be construed as a potential conflict of interest.

## References

[1] Johansson MW Keyser P Sritunyalucksana K Söderhäll K Crustacean haemocytes and haematopoiesis. Aquaculture (1992) 191:0–52. 10.1016/S0044-8486(00)00418-X

[2] Söderhäll K Crustacean immunity. Annu Rev Fish Dis (1992) 2:3–23. 10.1016/0959-8030(92)90053-Z

[3] Wang XW Wang JX Diversity and multiple functions of lectins in shrimp immunity. Dev Comp Immunol (2013) 39:27–38. 10.1016/j.dci.2012.04.009 22561073

[4] Amparyup P Kondo H Hirono I Aoki T Tassanakajon A Molecular cloning, genomic organization and recombinant expression of a crustin-like antimicrobial peptide from black tiger shrimp *Penaeus monodon* . Mol Immunol (2008) 45:1085–93. 10.1016/j.molimm.2007.07.031 17850873

[5] Gan ZT The Role of Antimicrobial Peptides in Innate Immunity. Integr Comp Biol (2003) 43:300–4. 10.1093/icb/43.2.300 21680437

[6] Jiravanichpaisal P Lee BL Söderhäll K Cell-mediated immunity in arthropods: Hematopoiesis, coagulation, melanization and opsonization. Immunobiology (2006) 211:230–6. 10.1016/j.imbio.2005.10.015 16697916

[7] Li FH Xiang JH Recent advances in researches on the innate immunity of shrimp in China. Dev Comp Immunol (2013) 39:11–26. 10.1016/j.dci.2012.03.016 22484214

[8] Söderhäll K Smith VJ Separation of the haemocyte populations of *Carcinus Maenas* and other marine decapods, and prophenoloxidase distribution. Dev Comp Immunol (1983) 7:229–39. 10.1016/0145-305X(83)90004-6 6409683

[9] Yao CL Li FH Xiang JH Crustacean haemocytes and their function in immune responses. Zoological Res (2006) 27:549–57.

[10] Yang CC Lu CL Chen S Liao WL Chen SN Immune gene expression for diverse haemocytes derived from pacific white shrimp, Litopenaeus vannamei. Fish Shellfish Immunol (2015) 44:265–71. 10.1016/j.fsi.2015.02.001 25681751

[11] Xing J Chang YH Tang XQ Sheng XZ Zhan WB Separation of haemocyte subpopulations in shrimp *Fenneropenaeus chinensis* by immunomagnetic bead using monoclonal antibody against granulocytes. Fish Shellfish Immunol (2017) 60:114–8. 10.1016/j.fsi.2016.11.034 27847341

[12] Zhu L Chang YH Xing J Tang XQ Sheng XZ Zhan WB Comparative proteomic analysis between two haemocyte subpopulations in shrimp *Fenneropenaeus chinensis* . Fish Shellfish Immunol (2018) 72:325–33. 10.1016/j.fsi.2017.09.074 28966142

[13] Zhu L Tang XQ Xing J Sheng XZ Zhan WB Differential proteome of haemocyte subpopulations responded to white spot syndrome virus infection in Chinese shrimp *Fenneropenaeus chinensis* . Dev Comp Immunol (2018) 84:82–93. 10.1016/j.dci.2018.02.003 29427599

[14] Kobayashi M Johansson MW Söderhäll K The 76 kD cell-adhesion factor from crayfish haemocytes promotes encapsulation in vitro. Cell Tissue Res (1990) 260:13–8. 10.1007/BF00297485

[15] Vargas-Albores F Gollas-Galván T Hernández-López J Functional characterization of Farfantepenaeus californiensis, Litopenaeus vannamei and L. stylirostris haemocyte separated using density gradient centrifugation. Aquaculture Res (2015) 36:352–60. 10.1111/j.1365-2109.2004.01207.x

[16] Omori SA Martin GG Hose JE Morphology of hemocyte lysis and clotting in the ridgeback prawn, *Sicyonia ingentis* . Cell Tissue Res (1989) 255:117–23. 10.1007/BF00229072

[17] Zhang YW Lai HWL Yew DT Apoptosis–a brief review. Neuroembryol Aging (2004) 05:47–59. 10.1159/000085404

[18] Williams GT Programmed cell death: apoptosis and oncogenesis. Cell (1991) 65:1097–8. 10.1016/0092-8674(91)90002-g 1648446

[19] Everett H Mcfadden G Apoptosis: an innate immune response to virus infection. Trends Microbiol (1999) 7:160–5. 10.1016/S0966-842X(99)01487-0 10217831

[20] Clarke TE Clem RJ Insect defenses against virus infection: the role of apoptosis. Int Rev Immunol (2003) 22:401–24. 10.1080/08830180305215 12959752

[21] Hay S Kannourakis G A time to kill: Viral manipulation of the cell death program. J Gen Virol (2002) 83:1547–64. 10.1099/0022-1317-83-7-1547 12075073

[22] Latif RS Wu JL Wang HV Hong JR Aquatic viruses induce host cell death pathways and its application. Virus Res (2016) 211:133–44. 10.1016/j.virusres.2015.10.018 26494167

[23] Amara A Mercer J Viral apoptotic mimicry. Nat Rev Microbiol (2015) 13:461–9. 10.1038/nrmicro3469 PMC7097103 26052667

[24] Best SM Bloom ME Caspase activation during virus infection: more than just the kiss of death? Virology (2004) 320:191–4. 10.1016/j.virol.2003.11.025 PMC7126475 15053016

[25] Best SM Viral Subversion of Apoptotic Enzymes: Escape from Death Row. Annu Rev Microbiol (2008) 62:171–92. 10.1146/annurev.micro.62.081307.163009 PMC2562643 18729734

[26] Stentiford GD Neil DM Peeler EJ Shields JD Small HJ Flegel TW Disease will limit future food supply from the global crustacean fishery and aquaculture sectors. J Invertebrate Pathol (2012) 110:141–57.23. 10.1016/j.jip.2012.03.013 22434002

[27] Rajan PR Ramasamy P Purushothaman V Brennan GP White spot baculovirus syndrome in the Indian shrimp Penaeus monodon and P. indicus. Aquaculture (2000) 184:0–44. 10.1016/S0044-8486(99)00315-4

[28] Wongprasert K Khanobdee K Glunukarn SS Meeratana P Withyachumnarnkul B Time-course and levels of apoptosis in various tissues of black tiger shrimp *Penaeus monodon* infected with white-spot syndrome virus. Dis Aquat Organisms (2003) 55:3–10. 10.3354/dao055003 12887248

[29] Chen J Gong Y Zheng H Ma H Aweya JJ Zhang Y SpBcl2 promotes WSSV infection by suppressing apoptotic activity of hemocytes in mud crab, *Scylla paramamosain* . Dev Comp Immunol (2019) 100:103421. 10.1016/j.dci.2019.103421 31254562

[30] Yuan FH Chen YG Zhang ZZ Yue HT Bi HT Yuan K Down-regulation apoptosis signal-regulating kinase 1 gene reduced the Litopenaeus vannamei hemocyte apoptosis in WSSV infection. Fish Shellfish Immunol (2016) 50:109–16. 10.1016/j.fsi.2015.12.003 26806164

[31] Rijiravanich ABCL Withyachumnarnkul B Knocking down caspase-3 by RNAi reduces mortality in Pacific white shrimp Penaeus (*Litopenaeus*) *vannamei* challenged with a low dose of white-spot syndrome virus. Fish Shellfish Immunol (2008) 24:308–13. 10.1016/j.fsi.2007.11.017 18248799

[32] Leu JH Lin SJ Huang JY Chen TC Lo CF A model for apoptotic interaction between white spot syndrome virus and shrimp. Fish Shellfish Immunol (2013) 34:1011–7. 10.1016/j.fsi.2012.05.030 22683516

[33] Zhang XB Wang L Zhi B Wu W Requirement for shrimp caspase in apoptosis against virus infection. Dev Comp Immunol (2008) 32:706–15. 10.1016/j.dci.2007.10.010 18068223

[34] He F Fenner BJ Godwin AK Kwang J White Spot Syndrome Virus Open Reading Frame 222 Encodes a Viral E3 Ligase and Mediates Degradation of a Host Tumor Suppressor via Ubiquitination. J Virol (2006) 80:3884–92. 10.1128/JVI.80.8.3884-3892.2006 PMC1440444 16571805

[35] Tang XQ Cui C Liang QR Sheng XZ Xing J Zhan WB Apoptosis of hemocytes is associated with the infection process of white spot syndrome virus in *Litopenaeus vannamei* . Fish Shellfish Immunol (2019) 94:907–15. 10.1016/j.fsi.2019.10.017 31604147

[36] Wang YT Liu W Seah JN Lam CS Xiang JH Korzh V White spot syndrome virus (WSSV) infects specific hemocytes of the shrimp P*enaeus merguiensis* . Dis Aquat Organisms (2002) 52:249. 10.3354/dao052249 12553452

[37] Jiravanichpaisal P Sricharoen S Söderhäll K White spot syndrome virus (WSSV) interaction with crayfish haemocytes. Fish Shellfish Immunol (2006) 20:718–27. 10.1016/j.fsi.2005.09.002 16260153

[38] Lo CF Leo JH Ho CH Chen CH Peng SE Chen YT Detection of baculovirus associated with white spot syndrome (WSBV) in penaeid shrimps using polymerase chain reaction. Dis Aquat Organisms (1996) 25:133–41. 10.3354/dao025133

[39] Yuan L Zhang X Chang M Jia C Hemmingsen SM Dai H A new fluorescent quantitative PCR-based in vitro neutralization assay for white spot syndrome virus. J Virol Methods (2007) 146:96–103. 10.1016/j.jviromet.2007.06.009 17645951

[40] Sahtout AH Hassan MD Shariff M DNA fragmentation, an indicator of apoptosis, in cultured black tiger shrimp *Penaeus monodon* infected with white spot syndrome virus (WSSV). Dis Aquat Organisms (2001) 44:155–9. 10.3354/dao044155 11324818

[41] Feng JX Tang XQ Zhan WB Analysis and identification of tyrosine phosphorylated proteins in hemocytes from *Fenneropenaeus chinensis* (Decapoda: Penaeidae) infected with white spot syndrome virus. J Crustacean Biol (2014) 34:453–9. 10.1163/1937240X-00002241

[42] Wang YN Zhan WB Xing J Jiang YS In vivo neutralization assays by monoclonal antibodies against white spot syndrome virus in crayfish (*Cambarus proclarkii*). Acta Oceanol Sin (2018) 2:131–7.

[43] Jiang YS Zhan WB Sheng XZ Neutralizing assay of monoclonal antibodies against white spot syndrome virus (WSSV). Aquaculture (2007) 272:216–22. 10.1016/j.aquaculture.2007.07.229

[44] Zhang L Tang XQ Sheng XZ Zhan WB Analysis on Dynamic Changes of WSSV Amount in *Procambarus clarkii* through Quantitative Real-time PCR. China Anim Health Inspec (2015) 32:69–74. 10.1016/j.aquaculture.2007.07.229

[45] Zhan WB Wei XL Xing J Zhang ZD Characterization of monoclonal antibodies to haemocyte types of the shrimp, *Fenneropenaeus chinensis* . Crustaceana (2008) 81:931–42. 10.1163/156854008X354993

[46] Lin YB Zhan WB Li Q Zhang ZD Wei XM Sheng XZ Ontogenesis of haemocytes in shrimp (*Fenneropenaeus chinensis*) studied with probes of monoclonal antibody. Dev Comp Immunol (2007) 31:1073–81. 10.1016/j.dci.2007.02.001 17428538

[47] Flegel TW Pasharawipas T Active viral accommodation: a new concept for crustacean response to viral pathogens. Adv Shrimp Biotechnol (1998) 245–50.

[48] Rhee WJ Park TH Flow cytometric analysis of the effect of silkworm hemolymph on the baculovirus-induced insect cell apoptosis. J Microbiol Biotechnol (2001) 11:853–7.

[49] Khanobdee K Soowannayan C Flegel TW Ubol S Withyachumnarnkul B Evidence for apoptosis correlated with mortality in the giant black tiger shrimp *Penaeus monodon* infected with yellow head virus. Dis Aquat Organisms (2002) 48:79–90. 10.3354/dao048079 12005239

[50] Hardwick MJ Apoptosis in viral pathogenesis. Cell Death Differ (2001) 8(2):109–10. 10.1038/sj.cdd.4400820 11313711

[51] Zhou X Jiang W Liu Z Liu S Liang X Virus infection and death receptormediated apoptosis. Viruses (2017) 9(11):316. 10.3390/v9110316 PMC5707523 29077026

[52] Silke A Hösel M Protzer U Apoptosis of hepatitis B virus-infected hepatocytes prevents release of infectious virus. J Virol (2010) 84(22):11994–2001. 10.1128/JVI.00653-10 PMC2977891 20719950

[53] Ampomah PB Lina HK Influenza A virus-induced apoptosis and virus propagation. Apoptosis (2020) 25.1:1–11. 10.1007/s10495-019-01575-3 31667646

[54] Goldmacher VS Bartle LM Skaletskaya A Dionne CA Kedersha NL Vater CA A cytomegalo virus encoded mitochondria-localized inhibitor of apoptosis structurally unrelated to Bcl-2. Proc Natl Acad Sci (1999) 96(22):12536–41. 10.1073/pnas.96.22.12536 PMC22976 10535957

[55] Kvansakul M van Delft MF Lee EF Gulbis JM Fairlie WD Huang DC A structural viral mimic of prosurvival Bcl-2: a pivotal role for sequestering proapoptotic Bax and Bak. Mol Cell (2007) 25(6):933–42. 10.1016/j.molcel.2007.02.004 17386268

[56] Tarodi B Subramanian T Chinnadurai G Epstein-Barr virus BHRF1 protein protects against cell death induced by DNA-damaging agents and heterologous viral infection. Virology (1994) 201(2):404–7. 10.1006/viro.1994.1309 8184552

[57] Barrera-Vázquez OS Cancio-Lonches C Hernández-González O Chávez-Munguia B Villegas-Sepúlveda N Gutiérrez-Escolano AL The feline calicivirus leader of the capsid protein causes survivin and XIAP downregulation and apoptosis. Virology (2019) 527:146–58. 10.1016/j.virol.2018.11.017 30529563

[58] Garant KA Shmulevitz M Pan L Daigle RM Ahn DG Gujar SA Oncolytic reovirus induces intracellular redistribution of Ras to promote apoptosis and progeny virus release. Oncogene (2016) 35(6):771–82. 10.1038/onc.2015.136 25961930

[59] Yuan S Zhang N Xu L Zhou L Ge X Guo X Induction of apoptosis by the nonstructural protein 4 and 10 of porcine reproductive and respiratory syndrome virus. PloS One (2016) 11(6):e0156518. 10.1371/journal.pone.0156518 27310256 PMC4911139

[60] Flegel TW Update on viral accommodation, a model for host-viral interaction in shrimp and other arthropods. Dev Comp Immunol (2007) 31:217–31. 10.1016/j.dci.2006.06.009 16970989

[61] Yan F Xia D lv S Qi Y Xu H Functional analysis of the orf390 gene of the White Spot Syndrome Virus. Virus Res (2010) 151:39–44. 10.1016/j.virusres.2010.03.014 20362018

[62] Leu JH Chen LL Lin YR Kou GH Lo CF Molecular mechanism of the interactions between white spot syndrome virus anti-apoptosis protein AAP-1 (WSSV449) and shrimp effector caspase. Dev Comp Immunol (2010) 34:1068–74. 10.1016/j.dci.2010.05.010 20546774

[63] Wang HC Ko TP Lee YM Leu JH Ho CH Huang WP White spot syndrome virus protein ICP11: A histone-binding DNA mimic that disrupts nucleosome assembly. Proc Natl Acad Sci U States America (2008) 105:20758–63. 10.1073/pnas.0811233106 PMC2605418 19095797

[64] Wang PH Huang T Zhang X He JG Antiviral defense in shrimp: From innate immunity to viral infection. Antiviral Res (2014) 108:129–41. 10.1016/j.antiviral.2014.05.013 24886688

[65] Sun YM Li FH Xiang JH Analysis on the dynamic changes of the amount of WSSV in Chinese shrimp *Fenneropenaeus chinensis* during infection. Aquaculture (2013) 376:124–32. 10.1016/j.aquaculture.2012.11.014

[66] Li C Weng S He JG WSSV–host interaction: Host response and immune evasion. Fish Shellfish Immunol (2018) 84:558–71. 10.1016/j.fsi.2018.10.043 30352263

[67] Roulston A Marcellus RC Branton PE Viruses and Apoptosis. J Gen Virol (1999) 53:577–628. 10.1146/annurev.micro.53.1.577 10547702

[68] Wu JL Muroga K Apoptosis does not play an important role in the resistance of ‘immune’ *Penaeus japonicus* against white spot syndrom virus. J Fish Dis (2004) 27:15–21. 10.1046/j.1365-2761.2003.00491.x 14986935

[69] Badley AD Pilon AA Landay A Lynch DH Mechanisms of HIV-associated lymphocyte apoptosis. Blood (2000) 96:2951–64. 10.1182/blood.V96.9.2951.h8002951_2951_2964 11049971

[70] Ahr B Robert-Hebmann V Devaux C Biard-Piechaczyk M Apoptosis of uninfected cells induced by HIV envelope glycoproteins. Retrovirology (2004) 1:12. 10.1186/1742-4690-1-12 15214962 PMC446229

[71] Okada H Kobune F Sato TA Kohama T Takeuchi Y Abe T Extensive lymphopenia due to apoptosis of uninfected lymphocytes in acute measles patients. Arch Virol (2000) 145:905–20. 10.1007/s007050050683 10881678

[72] Bosnjak L Miranda-Saksena M Koelle DM Boadle RA Jones CA Cunningham AL Herpes Simplex Virus Infection of Human Dendritic Cells Induces Apoptosis and Allows Cross-Presentation via Uninfected Dendritic Cells. J Immunol (2005) 174:2220–7. 10.4049/jimmunol.174.4.2220 15699155

[73] Linda D Pedro SC Inmaculada G Covadonga A Investigations of Pro- and Anti-Apoptotic Factors Affecting African Swine Fever Virus Replication and Pathogenesis. Viruses (2017) 9:241. 10.3390/v9090241 PMC5618007 28841179

[74] Li M Li J Yang J Liu J Zhang Z Song X RSV replication is promoted by autophagy-mediated inhibition of apoptosis. J Virol (2018) 92:02193. 10.1128/JVI.02193-17 PMC5874425 29386287

[75] Li F Chang X Xu L Yang F Different roles of crayfish hemocytes in the uptake of foreign particles. Fish Shellfish Immunol (2018) 77:112–9. 10.1016/j.fsi.2018.03.029 29578050

[76] Du J Zhu H Ren Q Liu P Chen J Xiu Y Flow cytometry studies on the *Macrobrachium rosenbergii* hemocytes sub-populations and immune responses to novel pathogen spiroplasma MR-1008. Fish Shellfish Immunol (2012) 33:795–800. 10.1016/j.fsi.2012.07.006 22842149

[77] Xian JA Zhang XX Wang DM Li JT Zheng PH Lu YP Various cellular responses of different shrimp haemocyte subpopulations to lipopolysaccharide stimulation. Fish Shellfish Immunol (2017) 69:195–9. 10.1016/j.fsi.2017.08.025 28842372

[78] Havanapan PO Taengchaiyaphum S Ketterman AJ Krittanai C Yellow head virus infection in black tiger shrimp reveals specific interaction with granule-containing hemocytes and crustinPm1 as a responsive protein. Dev Comp Immunol (2016) 54:126–36. 10.1016/j.dci.2015.09.005 26384157

[79] Liu CH Cheng W Chen JC The peroxinectin of white shrimp *Litopenaeus vannamei* is synthesised in the semi-granular and granular cells, and its transcription is up-regulated with *Vibrio alginolyticus* infection. Fish Shellfish Immunol (2005) 18:431–44. 10.1016/j.fsi.2004.10.005 15683919

[80] Ding ZF Du J Ou JT Li WJ Wu T Xiu YJ Classification of circulating hemocytes from the red swamp crayfish *Procambarus clarkii* and their susceptibility to the novel pathogen *Spiroplasma eriocheiris in* vitro. Aquaculture (2012) 356:371–80. 10.1016/j.aquaculture.2012.04.042

