# Supplementary material for: Differential Apoptotic Responses of Hemocyte Subpopulations to White Spot Syndrome Virus Infection in Fenneropenaeus chinensis
Source: Front Immunol. 2020 Dec 7;11:594390. doi: 10.3389/fimmu.2020.594390 (PMC7750459; doi:10.3389/fimmu.2020.594390)
Supplement: Supplementary file 1 [file DataSheet_1.docx]

**Supplementary material**

**Table S1 The change of the total hemocytes density in hemolymph post-WSSV infection**

|  | WSSV-infection group  Total hemocytes  (x10^6^ cell per mL) | PBS-injection group  Total hemocytes  (x10^6^ cell per mL) |
| --- | --- | --- |
| 0 hpi | 11.72±0.50 | 11.68±0.45 |
| 6 hpi | 11.35±0.61 | 11.49±0.63 |
| 12 hpi | 10.02±0.57 | 11.55±0.50 |
| 18 hpi | 9.00±0.66 | 11.62±0.49 |
| 24 hpi | 4.77±0.60 | 11.46±0.40 |
| 36 hpi | 2.93±0.45 | 11.29±0.58 |
| 48 hpi | 1.01±0.40 | 11.20±0.50 |
| 60 hpi | 0.75±0.32 | 11.03±0.46 |
| 72 hpi | 0.66±0.36 | 11.12±0.34 |

Values are means ± SD (*n* = 3).


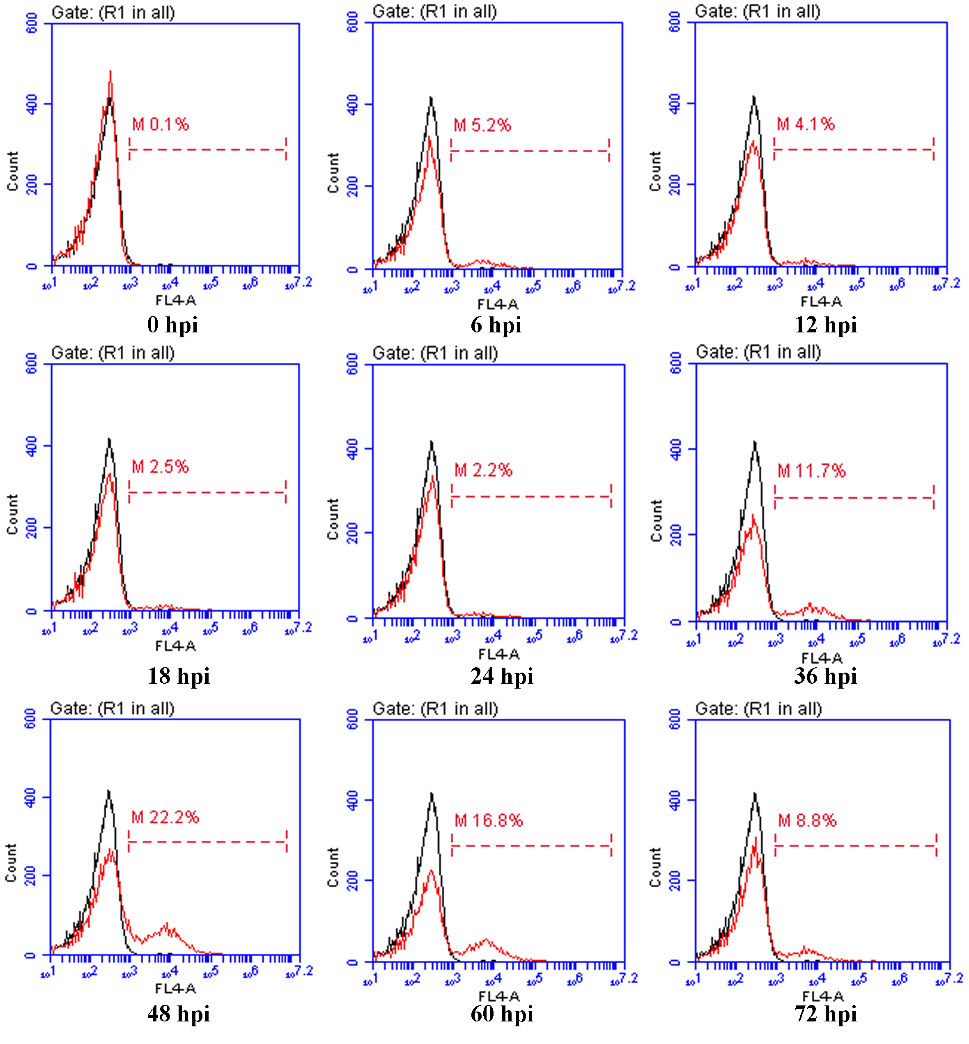
**Figure.S1 Analysis of the apoptotic dynamic of hemocytes in F. chinensis post-WSSV infection by flow cytometry.**


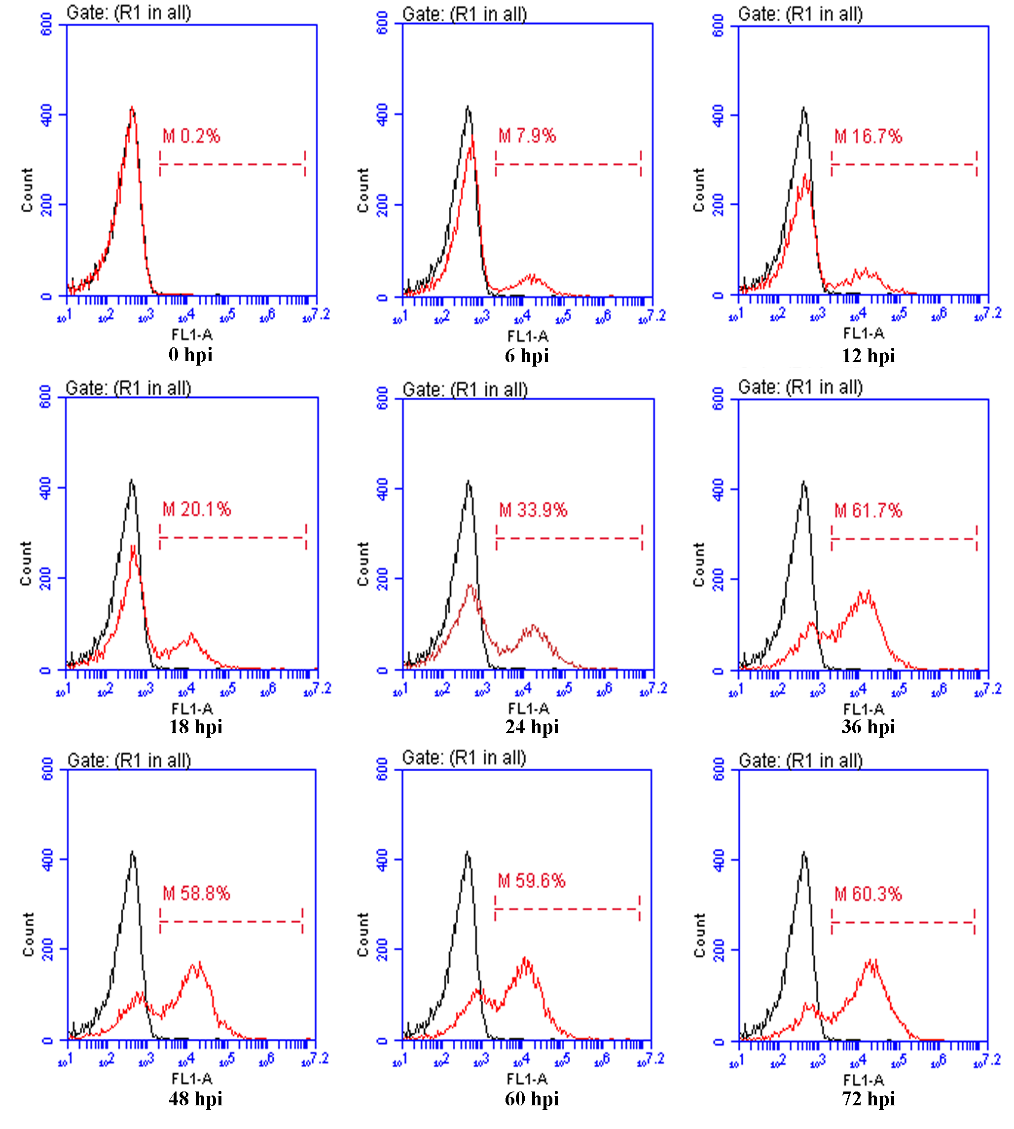
**Figure.S2 Analysis of the infected dynamics of hemocytes in *F. chinensis* post-WSSV infection by flow cytometry.**

**Table S2 The change of the granulocytes density post-WSSV infection**

|  | WSSV-infection group  Granulocytes  (x10^6^ cell per mL) | PBS-injection group  Granulocytes  (x10^6^ cell per mL) |
| --- | --- | --- |
| 0 hpi | 8.86±0.45 | 8.88±0.18 |
| 12 hpi | 6.98±0.34 | 8.37±0.31 |
| 24 hpi | 2.26±0.31 | 8.73±0.21 |
| 36 hpi | 1.29±0.25 | 8.30±0.27 |
| 48 hpi | 0.38±0.07 | 8.42±0.25 |
| 60 hpi | 0.25±0.05 | 7.29±0.13 |
| 72 hpi | 0.14±0.03 | 8.26±0.18 |

Values are means ± SD (*n* = 3).

**Table S3 The change of the hyalinocytes density post-WSSV infection**

|  | WSSV-infection group  Hyalinocytes  (x10^6^ cell per mL) | PBS-injection group  Hyalinocytes  (x10^6^ cell per mL) |
| --- | --- | --- |
| 0 hpi | 2.84±0.40 | 2.87±0.26 |
| 12 hpi | 3.24±0.30 | 3.18±0.17 |
| 24 hpi | 2.44±0.32 | 2.73±0.13 |
| 36 hpi | 1.63±0.39 | 2.99±0.16 |
| 48 hpi | 0.62±0.21 | 2.78±0.24 |
| 60 hpi | 0.50±0.24 | 3.08±0.28 |
| 72 hpi | 0.52±0.06 | 2.86±0.21 |

Values are means ± SD (*n* = 3).
